# Diagnostic and Interventional Sialendoscopy: A Four-Year Retrospective Study of 89 Patients

**DOI:** 10.3390/jcm14113938

**Published:** 2025-06-03

**Authors:** Iulian Filipov, Corina Marilena Cristache, Lucian Chirila, Mihai Sandulescu, Victor Nimigean

**Affiliations:** 1 Doctoral School, “Carol Davila” University of Medicine and Pharmacy, 37 Dionisie Lupu Street, 020021 Bucharest, Romania; iulian.filipov@drd.umfcd.ro; 2 Department of Dental Techniques, “Carol Davila” University of Medicine and Pharmacy, 8, Eroii Sanitari Blvd., 050474 Bucharest, Romania; 3 Department of Oral and Maxillofacial Surgery, Faculty of Dentistry, “Carol Davila” University of Medicine and Pharmacy, 19 Plevnei Ave., 010221 Bucharest, Romania; lucian.chirila@umfcd.ro; 4 Department of Implant-Prosthetic Therapy, Faculty of Dentistry, “Carol Davila” University of Medicine and Pharmacy, 050474 Bucharest, Romania; mihai.sandulescu@umfcd.ro; 5 Department of Anatomy, Faculty of Dentistry, “Carol Davila” University of Medicine and Pharmacy, 8, Eroii Sanitari Blvd., 050474 Bucharest, Romania; victor.nimigean@umfcd.ro

**Keywords:** sialendoscopy, salivary gland obstruction, sialolithiasis, ductal stenosis, submandibular gland, parotid gland, lithotripsy, minimally invasive surgery, gland preservation

## Abstract

**Background/Objectives:** Obstructive salivary gland disorders—primarily sialolithiasis and ductal stenosis—remain a significant source of morbidity, often requiring surgical intervention. Sialendoscopy has emerged as a minimally invasive, gland-preserving technique for both diagnosis and treatment. This retrospective study aimed to evaluate diagnostic and interventional sialendoscopy outcomes in a Romanian patient cohort and to identify gland-specific considerations in the management of salivary gland obstruction; **Methods:** A total of 89 patients with confirmed obstructive salivary gland disease (parotid or submandibular) were included. The most common indications included lithiasis, ductal stenosis, sialadenitis, and mixed pathologies; two cases of juvenile recurrent parotitis (JRP) were also managed. All patients underwent clinical evaluation, imaging (ultrasound, CBCT, CT, MR sialography), and sialendoscopic treatment between 2021 and 2025 in two centers. Data on demographics, imaging, calculus size, procedural technique, anesthesia, and complications were collected and analyzed using descriptive and inferential statistics; **Results:** The submandibular gland was more frequently involved (70.8%), with larger calculi compared to the parotid (mean 7.6 mm vs. 5.1 mm; *p* = 0.004). Minimally invasive techniques were predominantly used: sialolithotomy and intracorporeal lithotripsy were each performed in 32.6% of cases. Submandibulectomy was required in only 5.6% of patients. Most procedures (93.3%) were conducted under local anesthesia. Complication rates were low and primarily minor and self-limiting; **Conclusions:** Sialendoscopy is a safe and effective gland-preserving approach in managing obstructive salivary gland disorders. Gland-specific anatomy influences diagnostic pathways and therapeutic choices. These findings support broader adoption of sialendoscopy in routine practice and highlight the need for tailored management protocols based on gland involvement and stone characteristics. However, the study is limited by the absence of standardized post-intervention quality-of-life assessments and structured follow-up data on symptom recurrence.

## 1. Introduction

Obstructive disorders of the major salivary glands represent a significant clinical burden, frequently presenting as recurrent episodes of painful glandular swelling, often exacerbated during meals [1,2]. The most common etiologies are sialolithiasis (salivary stone formation) and ductal stenosis (narrowing of the salivary ducts), which together account for approximately 70% of obstructive salivary gland diseases [3,4]. While sialolithiasis is a well-known cause, recent population-based studies report a prevalence ranging from only 0.0014% to 0.0023% in the general population [5]. Moreover, its position as the leading cause of obstruction is under debate; emerging evidence suggests that ductal stenosis may be more prevalent, followed closely by sialolithiasis [4].

Other important causes of salivary obstruction include mucus plugs, papillary non-distensibility, and anatomical ductal variations [4]. Systemic conditions, such as Sjögren’s syndrome and juvenile recurrent parotitis, may further contribute to or predispose individuals to chronic obstructive sialadenitis by promoting ductal narrowing or altered salivary composition [6,7].

Clinically, these conditions result in impaired salivary flow, gland enlargement, and recurrent or chronic sialadenitis—a bacterial infection of the gland. If left untreated, such pathologies can significantly compromise patients’ quality of life and often necessitate therapeutic intervention [8].

Both the submandibular and parotid glands are commonly affected, although they differ in disease distribution and pathogenesis. Approximately 80–90% of salivary calculi form in the submandibular gland, likely due to its higher mucin and mineral content and the anatomical configuration of Wharton’s duct. In contrast, ductal strictures are more frequently observed in the parotid gland, with 70–75% of cases occurring in Stensen’s duct, often secondary to autoimmune conditions or prior infections [9].

Despite these anatomical and pathological differences, both glands are susceptible to recurrent obstruction and sialadenitis [10,11]. A comprehensive evaluation of obstructive salivary gland disorders must, therefore, account for gland-specific characteristics while maintaining a primary focus on relieving ductal obstruction and restoring normal salivary function.

Over the past two decades, sialendoscopy has become widely accepted as a minimally invasive, gland-preserving technique for the diagnosis and treatment of obstructive salivary gland disorders. Introduced in the early 1990s, the procedure involves inserting a miniature endoscope (typically 0.8–1.6 mm in diameter) through the natural ductal orifice, enabling direct, real-time visualization of the salivary ductal system [12,13,14,15]. Compared to traditional open surgery, which is associated with higher morbidity, including risks of nerve injury and xerostomia [16], sialendoscopy offers a safer, tissue-conserving alternative with significantly reduced complications [17,18].

The diagnostic value of sialendoscopy is further enhanced when combined with non-invasive imaging modalities such as ultrasound, cone-beam computed tomography (CBCT), and magnetic resonance (MR) sialography [19,20,21]. Ultrasound remains a valuable first-line tool for evaluating ductal dilation and stone presence. CBCT provides high-resolution anatomical detail and is particularly helpful in localizing radiopaque calculi, especially in the submandibular region [22]. MR sialography, on the other hand, offers superior soft tissue contrast and excels in visualizing intraparenchymal ducts and non-calcified obstructions. It is particularly advantageous in evaluating complex or suspected inflammatory strictures without the use of ionizing radiation [4].

The therapeutic capabilities of sialendoscopy have expanded with the development of instruments that allow for endoscopic removal of salivary stones, particularly those smaller than 7 mm in diameter, which can often be treated without the need for transoral sialolithotomy or gland excision [3]. For larger or impacted stones, sialendoscopy-assisted lithotripsy offers an effective solution by enabling stone fragmentation and extraction while preserving gland function [23,24,25]. In cases complicated by ductal stenosis or difficult access, adjunctive methods such as balloon dilation or stenting may be required [26,27]. Despite these technical challenges, the overall complication rate remains low, and sialendoscopy is increasingly regarded as the gold standard in the management of obstructive salivary gland diseases [28].

Accurate diagnosis is essential for guiding appropriate treatment. In this regard, diagnostic and interventional sialendoscopy serves as a **reference standard**, offering a direct and minimally invasive assessment of the salivary ductal system [27] and facilitating the identification of underlying pathologies such as calculi, strictures, mucous plugs, or anatomical anomalies [29].

While the efficacy of sialendoscopy has been demonstrated in multiple international studies [27,30], there remains a notable lack of data from Eastern Europe, including Romania, where clinical experience with this technique is still limited. In the absence of local evidence, traditional surgical excision continues to be widely practiced, despite the availability of less invasive, gland-sparing alternatives.

To address this gap, our retrospective study evaluates the clinical outcomes of diagnostic and interventional sialendoscopy in a cohort of 89 Romanian patients presenting with obstructive salivary gland disorders. This is one of the few studies conducted in Eastern Europe to analyze the use of sialendoscopy in clinical practice. Our objectives include assessing demographic and imaging data, procedural techniques, and therapeutic outcomes in order to support the broader implementation of this technique and enhance the quality of care for patients with salivary gland obstructions.

## 2. Materials and Methods

This retrospective clinical study was conducted in accordance with international ethical guidelines, including the Declaration of Helsinki, the Belmont Report, CIOMS-WHO guidelines, ICH-GCP standards, and EU Regulation 2016/679 (GDPR). Ethical approval was obtained from the Research Ethics Committee of the “Carol Davila” University of Medicine and Pharmacy, Bucharest (Approval No. 19772/2023). Written informed consent was obtained from all participants for both procedures and data collection.

The study included patients diagnosed with obstructive salivary gland disorders (parotid and/or submandibular glands) who underwent consultation and treatment between January 2021 and January 2025 in two centers affiliated with “Carol Davila” University of Medicine and Pharmacy from Bucharest, Romania: a stationary hospital unit and a private outpatient clinic, both equipped with diagnostic and therapeutic sialendoscopy facilities.

### 2.1. Inclusion and Exclusion Criteria

Patients were eligible if they

- Had a confirmed diagnosis of obstructive disorder of the major salivary glands (parotid or submandibular);
- Had complete demographic and clinical documentation, including sex, age, affected gland, diagnostic approach, treatment method, clinical evolution, and any complications.

A structured diagnostic protocol was applied to most patients, beginning with anamnesis and review of prior investigations, followed by soft tissue ultrasound, which was the primary imaging modality due to its non-invasive nature. When needed, cone-beam computed tomography (CBCT) was used to assess ductal pathology—particularly in suspected submandibular sialolithiasis—or to detect calculi not visualized by ultrasound. Magnetic resonance (MR) sialography was selectively employed in cases where ductal stenosis or extrinsic compression of Stensen’s duct was suspected. In rare instances, sialography–CBCT with contrast was used for cases with unclear etiology.

Patients were excluded if they

- Had systemic or locoregional conditions contraindicating sialendoscopy, such as hematologic disorders, coagulopathies, severe cardiovascular diseases, or systemic infections;
- Presented with acute suppurative sialadenitis or other high-risk comorbidities that significantly increased the potential for intra- or post-procedural complications.

### 2.2. Data Collection and Analysis

Data were extracted from patient medical records, operative reports, and follow-up documentation. For each patient, anamnesis data were reviewed, with particular attention paid to the onset, duration, and progression of symptoms, as well as a detailed description of associated clinical signs. Symptom duration was analyzed as a potential contributing factor to the severity and clinical presentation of obstructive salivary gland disease.

Clinical examination findings were documented, including visual inspection and bimanual palpation of the affected area. Evaluated structures included the facial soft tissues, overlying skin, oral mucosa, and salivary papilla. Bimanual palpation, especially in cases of submandibular lithiasis, often provided critical information for treatment planning. For instance, if a stone was palpable within Wharton’s duct, transoral sialolithotomy under endoscopic guidance was commonly indicated.

Diagnostic imaging data were also reviewed retrospectively.

Technical aspects of the procedure were evaluated based on operative reports.

Most procedures were conducted under local anesthesia, as documented in the medical records. Cases requiring general anesthesia were identified and justified based on the patient’s medical condition, psychological factors, or the complexity of the diagnostic or therapeutic procedure.

### 2.3. Statistical Analysis

All collected data were entered into Microsoft Excel and subsequently analyzed using IBM^®^ SPSS^®^ Statistics version 25.0 (IBM Corp., Armonk, NY, USA). Statistical analysis was conducted to explore associations between demographic variables, diagnostic strategies, therapeutic interventions, and clinical outcomes in patients undergoing salivary gland evaluation and treatment.

Descriptive statistics were used to summarize continuous variables such as patient age, number of calculi extracted, and stone size. These results were expressed as means and standard deviations (SD). Descriptive evaluations were stratified by gender and salivary gland involvement (parotid vs. submandibular).

Inferential statistical analysis included Welch’s *t*-test (independent-samples *t*-test) for continuous variables with unequal variances (e.g., age and calculus size comparisons by gender or gland type) and Chi-square (χ^2^) test to examine associations between categorical variables, such as gender, gland type, anesthesia use, diagnostic method, pathology type, and treatment approach.

Where appropriate, logistic regression models were attempted to predict binary outcomes (e.g., submandibulectomy as treatment failure), based on predictors including age, gender, calculus size, anesthesia, diagnostic tools, and pathology. However, perfect separation was detected in some models, limiting their reliability. Descriptive and inferential comparisons were therefore prioritized in these cases.

These methods enabled a structured evaluation of demographic characteristics, glandular distribution, diagnostic investigations, stone characteristics, and therapeutic modalities, contributing to the overall interpretation of clinical outcomes.

Statistical significance was defined as *p* < 0.05.

## 3. Results

**Figure 1:**
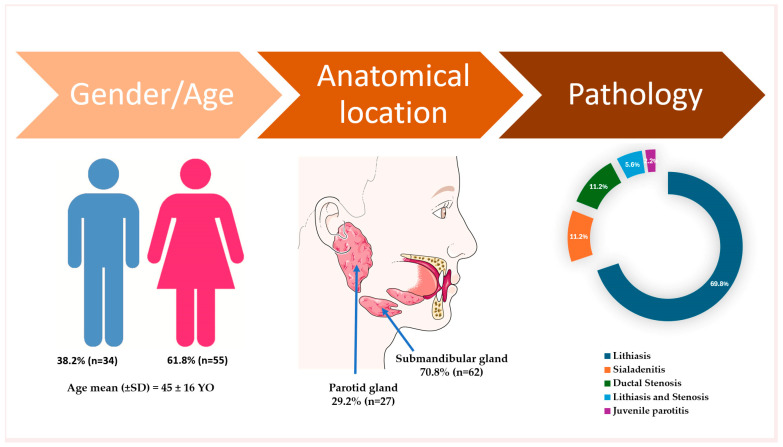
Descriptive data on demographic characteristics, gland involvement, and pathology in the included participants. The artwork used in this figure was adapted from Servier Medical Art (https://smart.servier.com/ accessed on 5 April 2025). Servier Medical Art by Servier is licensed under CC BY 4.0.

A total of 89 patients were included in this retrospective study. The mean age of the cohort was 45 ± 16 years (range: 14–78 years). Of the total participants, 61.8% were female and 38.2% were male (Figure 1). The follow-up period was between two months and four years, with a mean of 28 months. The stratification by gender and salivary gland involvement (parotid vs. submandibular) is presented in Table 1.

### 3.1. Gland Involvement

The submandibular gland was the most frequently affected site, accounting for 70.8% of all cases (Figure 1). When analyzed by gender, parotid gland involvement was observed in 32.7% of female patients and 26.5% of male patients, indicating a lower incidence of parotid involvement among males compared to females (Table 1).

### 3.2. Imaging Investigations

Various imaging modalities were employed to aid in diagnosis and assess the feasibility of calculus removal and were frequently used alongside diagnostic sialendoscopy.

Ultrasound was the most frequently employed, being performed in 77 patients (86.5%), both preoperatively and intraoperatively. It was used both prior to and during the initial consultation and showed consistent value across equipment and institutions (hospital/private clinic) in detecting obstructive pathology.

CBCT was performed in 54 patients (60.7%), with a statistically significant predominance in cases involving the submandibular gland (74.2% of all investigations), compared to 29.6% in parotid-related conditions (Table 1). CBCT proved valuable by providing essential anatomical detail for precise calculus localization and procedural planning.

Conventional medical CT was conducted in six patients (6.7%), with a statistically significant predominance in cases involving the parotid gland (Table 1). Although it provides superior image quality, its use in the evaluation of salivary gland obstruction is limited by the higher radiation dose compared to CBCT.

MR sialography was used in five patients (5.6%), primarily for non-lithiasic parotid obstruction.

CBCT Sialography was performed in three patients (3.4%), particularly when sialendoscopic access was not possible. It provided enhanced visualization of strictures or deep-seated calculi, aiding in treatment planning. Despite its diagnostic utility, its invasiveness and potential for allergic reaction were acknowledged as limitations [31].

### 3.3. Diagnosis and Pathology Distribution

The most prevalent pathology was sialolithiasis, observed in 64 patients (69.8%), with an additional 5 patients (5.6%) presenting with both lithiasis and ductal stenosis. Combined, lithiasic conditions accounted for over 75% of all cases. The submandibular gland was the most frequently affected site, with a statistically significant predominance compared to the parotid gland, which accounted for only 37.1% of all gland-related pathologies (Table 1).

Sialadenitis was identified in 10 patients (11.2%) (Figure 1). Among these, parotid gland involvement was observed in five patients, representing 18.5% of all parotid cases, while submandibular gland sialadenitis was recorded in five patients, corresponding to 8.1% of submandibular cases (Table 1).

Ductal stenosis, in the absence of calculi, was observed in 10 patients (11.2%), but when including patients with combined lithiasis and stenosis, the overall incidence of ductal stenosis was 16.8% (15 out of 89 patients) (Table 1).

### 3.4. Salivary Calculus Characteristics

The mean diameter of the salivary calculi was 7.0 ± 4.6 mm, with a range of 2 to 29 mm. In terms of stone burden:

- Sixty-one patients had a single calculus;
- Four patients had two calculi;
- Two patients had three calculi; and
- One patient presented with ten calculi.

For statistical purposes, the largest stone size per patient was recorded in the database.

### 3.5. Anesthesia

The vast majority of procedures (83 patients, 93.3%) were performed under local anesthesia. Benefits included reduced procedural cost, avoidance of general anesthesia-related risks, shorter recovery time, and the feasibility of conducting treatment in an outpatient setting. Only six patients (6.7%) required general anesthesia due to clinical or psychological considerations (Table 1).

### 3.6. Therapeutic Interventions

Diagnostic sialendoscopy was primarily performed using a 0.89 mm endoscope, which permitted access to second- and occasionally third-order ducts. For interventional procedures, endoscopes with diameters of 1.1 mm and 1.6 mm were used, with the narrower 1.1 mm scope preferred for the parotid (Stensen’s) duct. All procedures involved intermittent saline irrigation to maintain hydrostatic pressure, improve endoscope navigation, and clear the ductal lumen of fibrin debris.

Particular attention was paid to papilla instrumentation and endoscope insertion, which were frequently challenging. Lacrimal or metallic probes were typically used for ductal access, and magnification tools (4.5× surgical loupes or operating microscope) were employed in many cases to reduce the risk of complications such as false passage or trauma to peripapillary tissues.

The treatment strategy in this study emphasized minimally invasive, gland-preserving techniques. In line with structured decision-making approaches described in the literature, including the algorithmic model proposed by Foletti et al. [32], our management of salivary duct obstruction followed a practical framework guided by the affected gland and the anatomical location of the obstruction (distal, mid-duct, or proximal/hilar). In parotid gland cases, distal and mid-duct obstructions were treated endoscopically under local anesthesia, using dilation techniques. High-grade or fibrotic obstructions were managed with mechanical dilation, corticosteroid irrigation, and, in selected cases, temporary intraductal stenting to maintain ductal patency. Inflammatory-type obstructions responded well to lavage alone, while ductal dilatation associated with congenital or acquired megaduct required only conservative care, including gland massage and stimulation.

For submandibular gland obstruction, the treatment strategy was also location-dependent. Distal obstructions, particularly near the papilla, were often addressed by transoral ductal incision or marsupialization. Proximal and hilar obstructions, more challenging due to their deeper location, were typically managed via sialendoscopy with adjunctive balloon dilation, corticosteroid irrigation, and functional rehabilitation using sialogogues and gland massage. In complex cases with multifocal or extensive fibrotic obstructions, a combined endoscopic and transoral surgical approach was necessary. Across both glands, post-procedural management focused on preserving ductal patency and salivary flow, with follow-up assessments confirming symptomatic improvement in the majority of cases.

**Figure 2:**
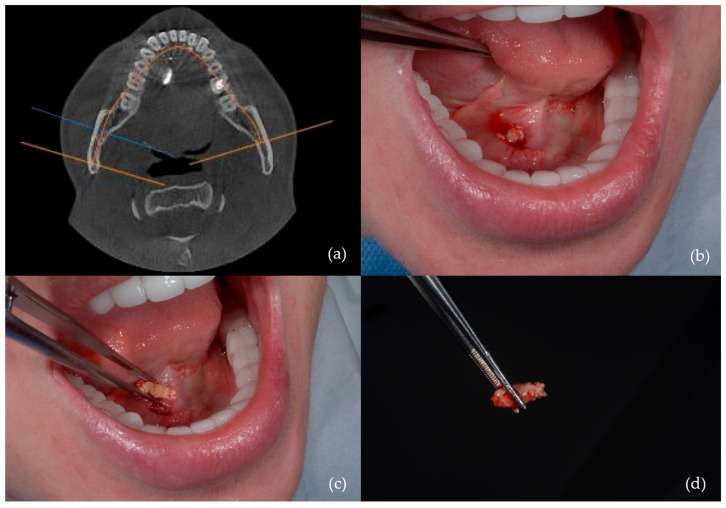
Sialolithotomy of a 9 mm calculus: (**a**) CBCT axial view showing the radiopaque calculus; (**b**) intraoperative view; (**c**) calculus removed from Wharton’s duct; (**d**) the calculus following sialolithotomy.

**Figure 3:**
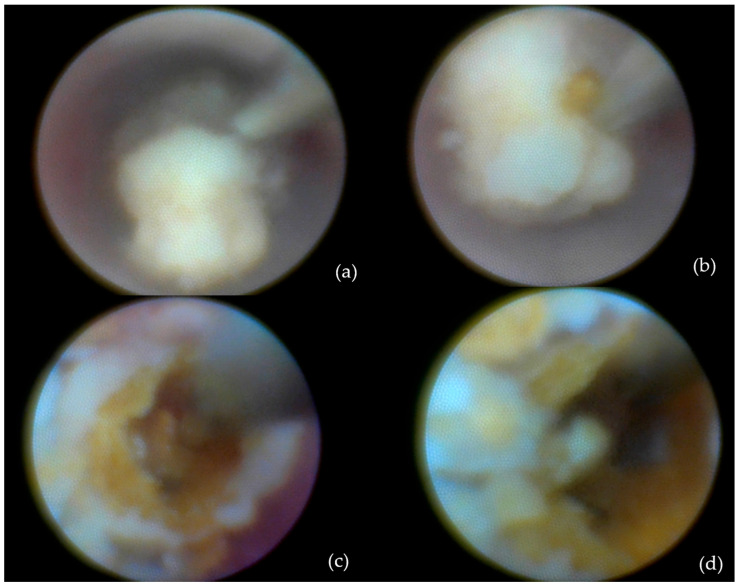
Calculus removal by sialolithotripsy—procedural steps: (**a**) Initial appearance; (**b**) identification of a longitudinal fissure; (**c**) appearance after removal of a fragment from the sialolith; (**d**) fragmentation of the sialolith into two distinct portions.

**Figure 4:**
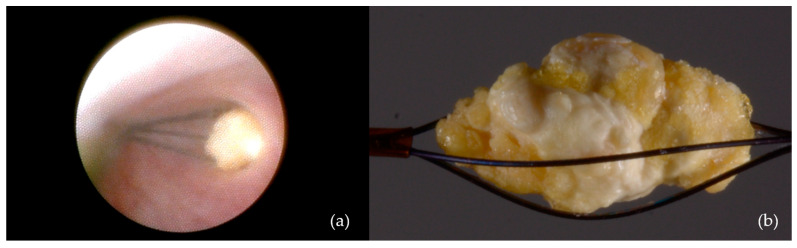
Interventional sialendoscopy using a Dormia basket: (**a**) Stone captured with the extraction probe; (**b**) stone secured within the Dormia-type device (macro lens view).

**Figure 5:**
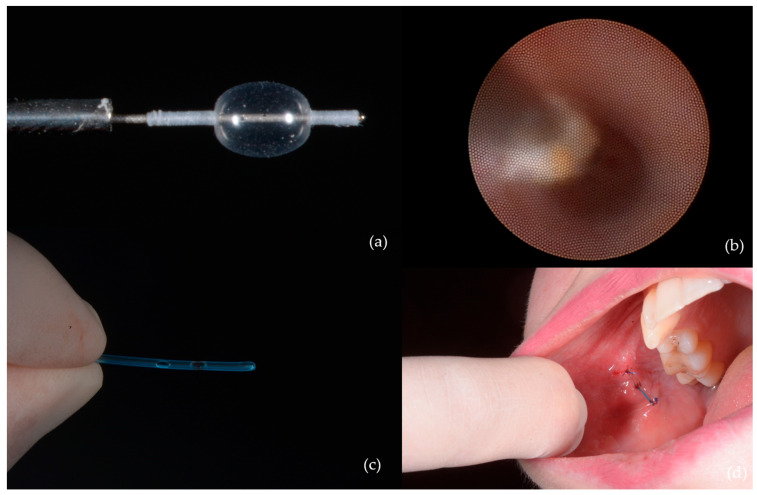
Ductal dilation and stenting: (**a**) Balloon used for dilation of the stenotic duct segment; (**b**) progressive ductal expansion; (**c**) stent used for maintaining ductal patency; (**d**) stent positioned and secured within Stensen’s duct.

**Figure 6:**
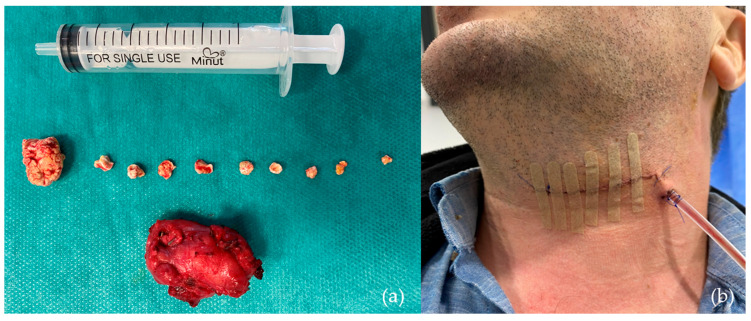
One of the cases requiring submandibulectomy involved a patient with 10 calculi: (**a**) Submandibular gland and sialoliths following submandibulectomy; (**b**) clinical aspect 48 h post-submandibulectomy.

The main interventions were as follows:

- Transoral sialolithotomy: Performed in 29 patients (32.6%) (Figure 2);

- Intracorporeal lithotripsy: Also in 29 patients (32.6%) (Figure 3);

- Dormia basket extraction: Used in eight patients (9%) (Figure 4);

- Ductal dilation and stenting were indicated in seven patients (7.9%), particularly in those with strictures or after papillotomy to enable removal of calculi (Figure 5);

- Submandibulectomy, as a last-resort surgical intervention (Figure 6), was conducted in only five patients (5.6%), typically in cases where minimally invasive techniques were unsuccessful or anatomical conditions precluded other options.

The use of sialolithotmy was significantly associated with submandibular gland (*p* < 0.001). Submandibulectomy was rare (n = 5); logistic regression indicated a trend toward higher odds in males, but it was not statistically significant (*p* = 0.06).

In this retrospective analysis, the recorded incidence of complications was low, supporting the favorable safety profile of the minimally invasive techniques employed. Documented complications were generally minor and self-limiting, with the most frequently reported being localized postoperative edema, moderate pain, and, in a few cases, secondary ductal infection. These infections responded well to oral antibiotic therapy with amoxicillin with clavulanic acid, 1 g, administered twice daily for five days. Importantly, no cases of iatrogenic ductal stenosis were identified in the reviewed records. Additionally, three cases of transient paresthesia were noted following transoral sialolithotomy, all of which resolved within a period of up to five months.

## 4. Discussion

To our knowledge, this is the first study published on a Romanian patient cohort evaluating the clinical utility of sialendoscopy as both a diagnostic and interventional tool for the management of obstructive salivary gland disorders.

In this retrospective analysis, we reviewed 89 cases of endoscopically assisted, transoral sialolith removal conducted over a four-year period. The mean age of the cohort was 45 ± 16 years, with 61.8% female predominance. These findings align with the prior literature that identifies peak incidence of sialolithiasis between the third and sixth decades of life [33,34]. Regarding gender distribution, some authors have reported a balanced ratio [33,35], while others have noted a male predominance [36]. The higher proportion of female patients may relate to hormonal changes associated with menopause, which contribute to xerostomia and altered salivary flow [37].

A meaningful comparison can be drawn with the multicenter study by Sánchez Barrueco et al. [4], which also reported a similar average patient age and female predominance. However, the pathology distribution differed notably: our study showed a predominance of lithiasis (69.8%), while theirs identified strictures (40.5%) as the leading etiology. This discrepancy may be influenced by imaging availability (particularly MR sialography), referral patterns, or the classification of emerging entities like lack of papilla distensibility.

In terms of gland involvement, submandibular cases were significantly more frequent (70.8%) compared to parotid involvement (29.2%). This finding is consistent with the previous literature citing the anatomical complexity of Wharton’s duct and the biochemical properties of submandibular saliva as factors contributing to a higher risk of lithiasis [1,38,39]. Calculi were more often found within the duct rather than in the hilum or intraglandular regions [39,40]. In contrast, parotid gland disease more often involved ductal strictures and typically required advanced imaging techniques.

Ultrasound was the primary diagnostic tool, used in 86.5% of patients. It remains the most accessible and cost-effective modality, especially effective in identifying sialolithiasis, although its sensitivity diminishes for strictures or non-calcified obstructions. CBCT, performed in 60.7% of cases, provided high-resolution, three-dimensional imaging, particularly valuable for submandibular lithiasis.

Each imaging modality used in the diagnostic work-up of chronic obstructive sialadenitis (COS) presents distinct strengths and limitations. Ultrasound offers high specificity but lower sensitivity for ductal strictures, particularly when not performed by experienced operators. CBCT offers excellent spatial resolution and is especially useful in submandibular cases for localizing radiopaque stones, though it lacks soft tissue contrast and involves radiation exposure. MR sialography, used in only 5.6% of patients due to limited availability and cost, remains the most sensitive and specific method for assessing soft tissue and intraparenchymal ductal pathology [4,41]. It was reserved for complex cases or when other methods were inconclusive. Ideally, a tiered diagnostic strategy—beginning with ultrasound and escalating to CBCT or MR sialography based on clinical suspicion—can enhance diagnostic accuracy while managing resources efficiently. Parotid gland involvement was more frequently associated with the need for advanced imaging modalities. This aligns with existing evidence highlighting the value of high-resolution imaging in parotid pathologies, as these techniques allow detailed visualization of the ductal anatomy—extending to second- and third-order branches—without requiring duct cannulation [42]. Future studies should aim to define clear imaging protocols tailored to specific clinical scenarios and gland involvement.

As for the distribution of pathology, lithiasis was the most frequent diagnosis (69.8%), followed by sialadenitis (11.2%), ductal stenosis (11.2%), and mixed lithiasis-stenosis cases (5.6%). These findings underscore the central role of calculi in obstructive salivary gland pathology in our region. In contrast, Sánchez Barrueco et al. reported a higher prevalence of strictures [4], possibly due to more frequent use of MR sialography and the inclusion of Lack of Papilla Distensibility (LCD). Although LPD is not a formal diagnostic index, it has recently been described as a relevant anatomical variant contributing to obstructive symptoms and difficult sialendoscope introduction, particularly in submandibular cases [4]. While not formally classified in our cohort, it may explain some cases of challenging ductal access without visible obstruction. Emerging etiologies such as mucus plugs and papillary non-distensibility, although not specifically captured in our cohort, are increasingly recognized as contributing causes [6,7].

In terms of salivary calculus characteristics, the average stone size was 7.6 mm in the submandibular gland and 5.1 mm in the parotid gland (*p* = 0.004), consistent with known anatomical and biochemical predispositions [33,35]. Larger stones were associated with more invasive procedures, and in rare cases (5.6%), submandibulectomy was necessary due to deep or multiple calculi. These observations support the need for individualized treatment planning based on stone size, number, and location.

In our cohort study, the overwhelming majority of sialendoscopic procedures (93.26%) were successfully performed under local anesthesia. The choice of anesthesia was based on preoperative clinical factors—such as systemic health conditions (e.g., dysautonomia), psychological considerations (e.g., significant anxiety), patient cooperation, and individual preference—rather than intraoperative findings or postoperative symptom resolution. Only 6.74% of patients required general anesthesia. This approach aligns with current evidence favoring local or conscious sedation for improved recovery time, lower cost, and comparable patient satisfaction [43,44]. Bawazeer et al. and Trujillo et al. found no significant differences in outcomes between general anesthesia and sedation techniques [43]. However, general anesthesia remains appropriate in specific scenarios, such as pediatric patients or those with complex intraglandular stones or limited cooperation [30,45].

In terms of treatment, transoral sialolithotomy and intracorporeal lithotripsy were each performed in 32.6% of cases. Additional techniques included Dormia basket extraction (9%) and ductal stenting (7.9%). Submandibulectomy was limited to 5.6% of cases and reserved for refractory situations. This stepwise, gland-preserving approach mirrors current best practices [10,46].

Sialendoscopy also demonstrated effectiveness in managing non-lithiasic obstructions, including Sjögren’s syndrome, juvenile recurrent parotitis, and suspected cases involving mucus plugs or papillary non-distensibility [6,7]. In our cohort, two patients (2.2%) were diagnosed with juvenile recurrent parotitis—a condition in which sialendoscopy may offer therapeutic benefit through ductal lavage, dilation, and intraductal corticosteroid administration [43,44]. Moreover, sialendoscopy is increasingly being explored as a minimally invasive treatment option in systemic conditions such as Sjögren’s syndrome, where it may help reduce glandular inflammation and improve ductal patency, thereby alleviating symptoms [45].

The stepwise, minimally invasive treatment protocol applied in our study was well tolerated and led to high rates of gland preservation. Complications were infrequent and generally minor.

Recent studies have highlighted that papilla typology may significantly influence procedural complexity and outcomes. Wharton’s duct has been classified into four types based on its macroscopic appearance, each associated with varying degrees of accessibility and risk of complications [47]. These insights emphasize the value of individualized planning and detailed preoperative assessment.

Lastly, logistic regression analysis in our study did not reveal any statistically significant predictors of treatment failure. However, trends suggested that male sex and the use of general anesthesia might be associated with an increased likelihood of submandibulectomy. These findings underscore the importance of careful patient selection and support the need for future prospective multicenter studies to improve risk stratification and optimize treatment algorithms.

Despite the valuable insights provided, this study has several limitations that should be explicitly acknowledged. First, although the cohort of 89 patients represents one of the largest Romanian series on sialendoscopy, it remains a relatively small sample size when stratified by pathology type, gland involvement, and treatment modality. This limitation may reduce the statistical power to detect subtle subgroup differences. Additionally, the low number of submandibulectomy cases (n = 5) limited the robustness of the logistic regression model and may have contributed to convergence issues and unstable estimates.

The retrospective design introduces inherent limitations, including potential selection and information biases. Data collection relied on available records, and long-term follow-up information was not uniformly documented for all patients. This affected the ability to assess recurrence rates and long-term complications in a standardized manner. Moreover, the study did not apply a clearly defined success criterion for treatment outcomes, and no standardized assessment of patient-reported outcomes or quality of life was included. These omissions limit the ability to evaluate the broader impact of sialendoscopy on functional recovery and patient well-being.

Another important limitation is the lack of consideration for systemic conditions, such as Sjögren’s syndrome or autoimmune diseases, that may influence the onset, progression, or recurrence of obstructive salivary gland disorders. Although some of these conditions were noted anecdotally, they were not consistently recorded or analyzed as part of the data set.

Future prospective, multicenter studies with larger and more diverse populations are warranted to validate these findings, define clear outcome measures, evaluate the influence of systemic disease, and incorporate quality-of-life assessments. These steps are essential to optimizing patient selection, standardizing care pathways, and refining therapeutic algorithms in clinical sialendoscopy practice.

## 5. Conclusions

This retrospective study is the first to evaluate the outcomes of diagnostic and interventional sialendoscopy in a Romanian patient cohort and adds valuable data to the limited literature available from Eastern Europe. The findings support the safety, feasibility, and clinical utility of minimally invasive techniques for the management of obstructive salivary gland disorders. Submandibular gland involvement was significantly associated with larger calculi and a greater need for invasive interventions, while parotid gland cases more often required advanced imaging.

Most procedures were successfully performed under local anesthesia, demonstrating the potential for efficient, cost-effective outpatient management. The low rate of reported complications and the limited need for submandibulectomy highlight the practicality of a personalized, gland-specific approach. These results advocate for the broader adoption of sialendoscopy as a first-line diagnostic and therapeutic tool in specialized salivary gland centers across the region.

## Figures and Tables

**Table 1:** Descriptive evaluations stratified by gender and salivary gland involvement (parotid vs. submandibular).

Variable	Female (n = 55)	Male (n = 34)	*p*-Value	Parotid	Submandibular	*p*-Value	Total (n = 89)
Age (mean ± SD)	43.7 ± 17.0	46.2 ± 14.8	0.46	41.9 ± 16.5	45.9 ± 16.0	0.29	45 ± 16
**Gland involved**							
Parotid	18 (32.7%)	9 (26.5%)	0.67	27 (29.2%)	-	-	27 (29.2%)
Submandibular	37 (67.3%)	25 (73.5%)	0.67	-	62 (70.8%)	-	62 (70.8%)
Calculus size (mm)	7.2 ± 4.2	6.8 ± 5.2	0.75	5.1 ± 1.8	7.6 ± 4.9	0.00 *	7.0 ± 4.6
**Pathology**							
Lithiasis	38 (69.1%)	24 (70.6%)	1.00	10 (37.1%)	52 (83.9%)	0.00 *	64 (69.8%)
Sialadenitis	7 (12.7%)	3 (8.8%)	0.83	5 (18.5%)	5 (8.1%)	0.28	10 (11.2%)
Ductal Stenosis	6 (10.9%)	4 (11.8%)	1.00	6 (22.2%)	4 (6.5%)	0.07	10 (11.2%)
Lithiasis and stenosis	4 (7.3%)	1 (2.9%)	0.69	4 (14.8%)	1 (1.5%)	0.05 *	5 (5.6%)
Juvenile parotitis	0	2 (5.9%)	0.28	2 (7.4%)	0	-	2 (2.2%)
**Imaging Investigations**							
Ultrasound	47 (85.5%)	30 (88.2%)	0.96	25 (92.6%)	52 (83.9%)	0.44	77(86.5%)
CBCT	34 (61.8%)	20 (58.8%)	0.95	8 (29.6%)	46 (74.2%)	0.00 *	54 (60.7%)
Medical CT	2 (3.6%)	4 (11.8%)	0.29	5 (18.5%)	1 (1.6%)	0.01 *	6 (6.7%)
MR Sialography	3 (5.5%)	2 (5.9%)	1.0	3 (11.11%)	2 (3.2%)	0.32	5 (5.6%)
CBCT Sialography	2 (3.6%)	1 (2.9%)	1.0	2 (7.4%)	1 (1.6%)	0.45	3 (3.4%)
**Anesthesia**							
Local	53 (96.4%)	30 (88.2%)	0.29	27 (100%)	56 (90.3%)	0.22	83 (93.3%)
General	2 (3.6%)	4 (11.8%)	0.29	0	6 (9.7%)	0.22	6 (6.7%)
**Therapeutic Interventions**							
Transoral sialolithotomy	19 (34.5%)	10 (29.4%)	0.79	0	29 (8.1%)	0.46	29 (32.6%)
Intracorporeal lithotripsy	18 (32.7%)	11 (32.4%)	1.0	12 (44.4%)	17 (27.4%)	0.18	29 (32.6%)
Dormia basket extraction	6 (10.9%)	2 (5.9%)	0.67	3 (11.1%)	5 (8.1%)	0.95	8 (9.0%)
Ductal dilation and stenting	4 (7.3%)	3 (8.8%)	1.0	4 (14.8%)	3 (4.8%)	0.24	7 (7.9%)
Submandibulectomy	1 (1.8%)	4 (11.8%)	0.13	0	5 (46.8%)	0.31	5 (5.6%)

CBCT = cone beam computed tomography; medical CT = medical computed tomography; MR = magnetic resonance imaging; * = statistically significant.

## Data Availability

Data supporting reported results are available from the corresponding author upon request.

## Abbreviations

The following abbreviations are used in this manuscript:

CBCT	Cone-beam computed tomography
COS	Chronic obstructive sialadenitis
JRP	Juvenile recurrent parotitis
LCD	Lack of Papilla Distensibility
Medical CT	Medical computed tomography
MR	Magnetic resonance
SD	Standard deviation
